# Nanosecond Pulsed Electric Fields Enhance the Anti-tumour Effects of the mTOR Inhibitor Everolimus against Melanoma

**DOI:** 10.1038/srep39597

**Published:** 2017-01-05

**Authors:** Jie Dai, Shan Wu, Yan Kong, Zhihong Chi, Lu Si, Xinan Sheng, Chuanliang Cui, Jing Fang, Jue Zhang, Jun Guo

**Affiliations:** 1 Key Laboratory of Carcinogenesis and Translational Research (Ministry of Education), Department of Renal Cancer and Melanoma, Peking University Cancer Hospital & Institute, Beijing, China; 2 College of Engineering, Peking University, Beijing, 100871, China; 3 Academy for Advanced Interdisciplinary Studies, Peking University, Beijing, 100871, China

## Abstract

The PI3K/mTOR/AKT pathway is activated in most melanomas, but mTOR inhibitors used singly have limited activity against advanced melanomas. The application of nanosecond pulsed electric fields (nsPEFs) is a promising cancer therapy approach. In this study, we evaluated the synergistic anti-tumour efficacy of the mTOR inhibitor everolimus in conjunction with nsPEFs against melanoma. The combined treatment of nsPEFs and everolimus gradually decreased cell growth concurrent with nsPEF intensity. nsPEFs alone or combined with everolimus could promote melanoma cell apoptosis, accompanied with a loss in cellular mitochondrial membrane potential and an increase in Ca^2+^ levels. *In vivo* experiments showed that a combination of the mTOR inhibitor everolimus and nsPEFs improved the inhibitory effect, and all skin lesions caused by nsPEFs healed in 1 week without any observed adverse effect. Combination treatment induced caspase-dependent apoptosis through the upregulation of the pro-apoptotic factor Bax and downregulation of the anti-apoptotic factor Bcl-2. Everolimus and nsPEFs synergistically inhibited angiogenesis by decreasing the expression of vascular endothelial growth factor (VEGF), VEGF receptor (VEGFR), and CD34. Our findings indicate that nsPEFs in combination with an mTOR inhibitor can be used as a potential treatment approach for advanced melanoma.

Metastatic melanoma is the most aggressive skin cancer, with a 5-year survival of less than 5% and a median survival of only 6–9 months^1^. The incidence of melanoma is increasing every year worldwide, and the American Cancer Society has estimated 76,380 new cases and 10,130 deaths from melanoma in the United States alone in 2016^2^.

Many exciting advancements have been achieved in the treatment of metastatic melanoma in the last 4 years, and targeted therapy has been demonstrated to be a powerful strategy to this end^3^,^4^,^5^. The mammalian target of rapamycin (mTOR) is a validated target in cancer treatment. The mTOR pathway has been demonstrated to be frequently hyper-activated in melanoma, resulting in increased cell proliferation and decreased cell apoptosis^6^,^7^,^8^. Everolimus (RAD001) is an inhibitor of mTOR, and it binds to FKBP12 and interacts with the mTOR complex, resulting in the inhibition of downstream signalling and growth suppression of tumour cells^9^,^10^. Everolimus can also inhibit the production of vascular endothelial growth factor (VEGF) and regulate angiogenesis^11^. Everolimus has been approved to treat HR+/HER2- advanced breast cancer, advanced neuroendocrine tumours of pancreatic origin, and advanced renal cell carcinoma^12^,^13^,^14^. However, a phase II trial of single-agent everolimus for the treatment of advanced melanoma failed, with a disease control rate of 29% and a progression-free survival (PFS) of 3 months^15^. Everolimus combined with chemotherapeutics or target therapeutic agents also could not prolong the survival of metastatic melanoma patients: the PFS of everolimus and temozolomide combination therapy was 2.4 months, while that of everolimus plus bevacizumab was 3.5 months^16^,^17^.

Nanosecond pulsed electric fields (nsPEFs) are characterized by ultra-short duration and ultra-high intensity electric fields. Typical nsPEFs have a duration of 60–300 ns, with a rise time of 4–30 ns^18^,^19^,^20^,^21^. Owing to its ultra-short duration and rise time, nsPEFs induce various biomedical effects that are clearly distinct from those of conventional electroporation. They have been reported to trigger various cellular responses, including phosphatidylserine translocation, cell membrane permeabilisation, and loss of mitochondrial membrane potential^22^,^23^,^24^,^25^. Besides, nsPEFs create little thermal effects when biological tissues or materials are treated^26^,^27^. In the treatment of solid tumours, nsPEFs have proved to be effective in inducing the growth inhibition of breast cancer and liver cancer^28^. Nuccitelli and Chen treated B16F10 murine melanoma with nsPEFs, and observed a rapid shrinkage in tumour cell nuclei and suppression of tumour blood flow^29^. nsPEFs have been demonstrated to be safe and effective in humans. The first human trial on nsPEFs was in a patient diagnosed with basal cell carcinoma, and pathological evaluation showed a complete remission 6 weeks after pulse delivery^30^. UCSF Benioff Children’s Hospital Oakland conducted the first clinical trial to treat skin cancer with nsPEFs (NCT01463709). Of 10 basal cell carcinomas treated, 7 became completely free of basaloid cells, 2 were partially ablated, and 1 recurred by week 10, with the appearance of squamous cell carcinoma^31^.

In addition to its use in monotherapy against solid tumours, the possibility of using nsPEFs as an adjuvant therapy has also been investigated. Recent studies showed that nsPEFs could enhance the efficacy of chemotherapeutic drugs *in vitro*, such as gemcitabine and pingyangmycin^32^,^33^,^34^. Thus, we speculate that nsPEFs might improve the therapeutic effect of the mTOR inhibitor everolimus in melanoma. In this study, we attempted to investigate the efficacy of the combination of everolimus and nsPEFs in melanoma cells, as well as in an orthotopic model, with the aim of developing a novel melanoma treatment approach.

## Results

### nsPEFs suppressed melanoma cell growth *in vivo* and *in vitro*

**Figure 1:**
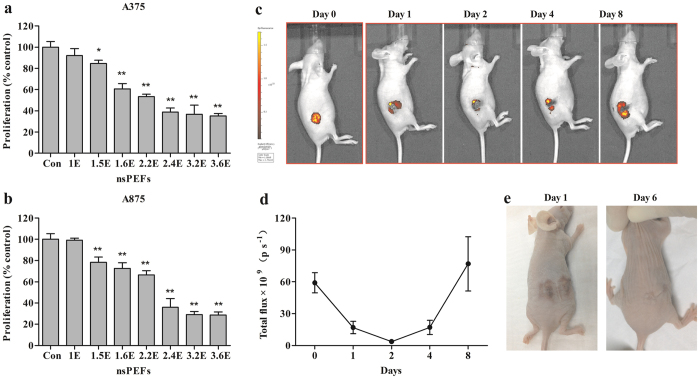
nsPEF treatment inhibited melanoma cell growth *in vivo* and *in vitro*. Effects of nsPEFs with high energy input in (**a**) A375 and (**b**) A875 cells. Pulse duration, 100 ns; electric field strength, 20–30 kV/cm; number of pulses delivered according to different energy levels, 10–100. The energy input of nsPEFs was calculated as energy input = (E^2^ × D^2^ × W × N)/(R × M), where E is the electric field strength (20–30 kV/cm); D, gap between electrodes (here, 2 mm); W, pulse duration (here, 100 ns); N, number of pulses (10–100 pulses); R, resistance in the cuvette with cells and suspending medium; and M, mass of the suspension in the cuvette. The specific parameters are listed in Supplementary Table S1. (**c**) Typical longitudinal monitoring of fluorescence images of tumour-bearing mice before and after nsPEF treatment. GFP activity in mice was detected by IVIS 200 before nsPEF treatment (day 0), as well as 1, 2, 4, and 8 days after nsPEF treatment. (**d**) Fluorescence intensities were quantified in photons per second. Ratios of mean ± SD (n = 5) were obtained. (**e**) Surface view after nsPEF treatment. The experiments were repeated thrice and yielded similar results. *P < 0.05, **P < 0.01 compared to control.

First, we evaluated the effect of nsPEFs on the growth of 4 melanoma cell lines (A375, A875, M21, and WM-115). Cells were treated with elevated energy input, and cell viability was detected after 72 h. Results showed that the cell proliferation could be reduced by nsPEFs, and the inhibitory effect was associated with nsPEF energy delivered to cells (Fig. 1a,b, and Supplementary Fig. S1). The inhibitory effect of nsPEFs was further detected *in vivo*. A375-GFP cells were inoculated into the flanks of BALB/c mice, and the Xenogen IVIS 200 system was used to longitudinally monitor the tumour development before and after nsPEF treatment. The fluorescence intensity of nsPEF-treated tumours decreased significantly, some of which even dropped to undetectable levels. However, portions of tumours showed a growth rebound after the first 3 days of remission (Fig. 1c and d). After nsPEF treatment, the skin showed local oedema and subcutaneous haemorrhage. Then, the skin begun to scab, and the lesions induced by nsPEFs healed in 1 week (Fig. 1e).

### nsPEFs and everolimus synergistically inhibited melanoma cell growth

**Figure 2:**
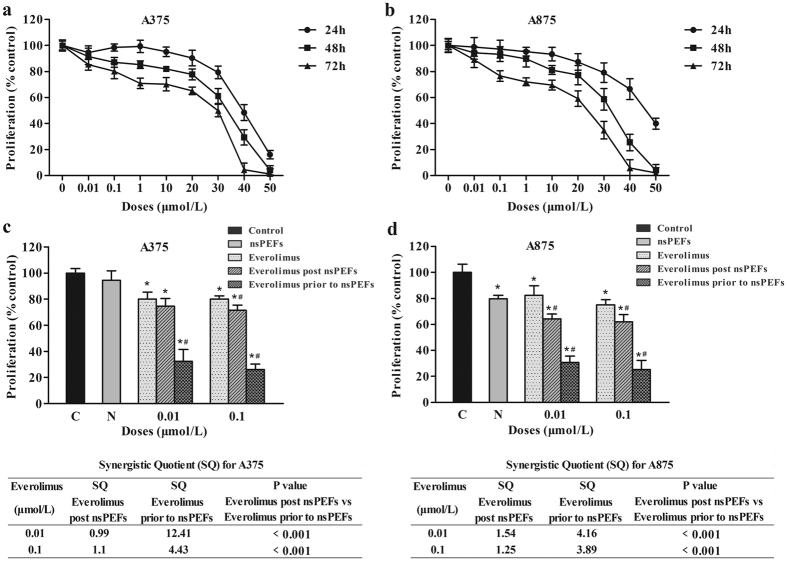
nsPEFs and everolimus synergistically inhibited melanoma cell growth *in vitro*. (**a**) and (**b**) Were effects of everolimus on the proliferation of melanoma cells *in vitro*. A375 and A875 cells were treated with elevated concentrations of everolimus for 24 h, 48 h, and 72 h. Cell viabilities were determined by CellTiter-Glo analysis. (**c**) and (**d**) Cells were first treated with nsPEFs, followed by incubation with everolimus for 48 h; or treated with everolimus for 48 h prior to nsPEF treatment. Synergism quotients (SQ) are shown for both treatment orders. Mean ± SD values of triplicate samples were determined. *P < 0.05 compared to control, ^#^P < 0.05 compared to everolimus single agent treatment.

The sensitivities of A375 and A875 cells to everolimus were evaluated. CellTiter-Glo analysis was performed to test the effect of everolimus on the proliferation of A375 and A875 cells. The cells were cultured in the presence of vehicle or elevated concentrations of everolimus for 24, 48, and 72 h. Neither cell line was sensitive to everolimus, the IC_50_ of both cells was >10 μM (Fig. 2a and b). To assess the inhibition effect of everolimus on mTOR-associated signalling mediators, we performed western blotting to analyse the phosphorylation of mTOR, S6, and 4EBP1. Cells were starved overnight and incubated for 1 h with control vehicle (DMSO) or everolimus. The results showed that everolimus could inhibit the phosphorylation of S6 but could not inhibit the phosphorylation of mTOR and 4EBP1 (Supplementary Fig. S2). We next examined the combination efficacy of nsPEFs and everolimus. Two combination strategies were investigated to determine the appropriate sequential method. One approach was to treat cells with nsPEFs first, followed by incubation with everolimus for 48 h. The other approach was treating cells with everolimus first, followed by nsPEF treatment. The synergistic quotient (SQ) was calculated to evaluate the combination effect. SQ was calculated according to the following formula: SQ = combination inhibition rate/(everolimus inhibition rate + nsPEF inhibition rate). An SQ value greater than 1 was considered to indicate a synergistic effect of the combination. As shown in Fig. 2c, nsPEF application before sequential treatment with everolimus showed the same inhibition trend as that of everolimus treatment alone in A375 cells, when A375 cells were subjected to nsPEFs after everolimus treatment, cell proliferation was significantly inhibited. nsPEFs in combination with everolimus exhibited statistically significant differences compared to everolimus treatment alone, implying that nsPEFs enhanced the efficacy of everolimus in the A375 cell line. A similar result was observed in A875 cells (Fig. 2d). Thus, nsPEF application following everolimus treatment was the best combination and sequence.

### nsPEF plus everolimus treatment promoted cell apoptosis

**Figure 3:**
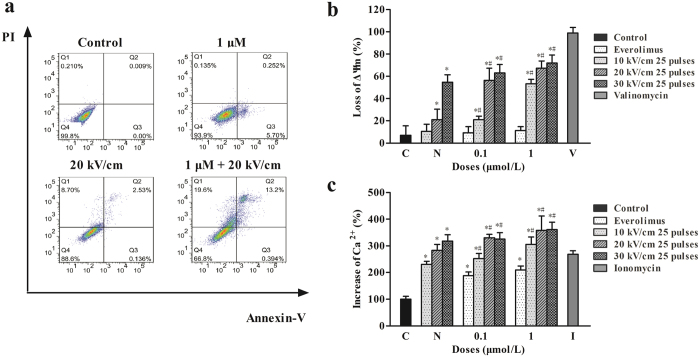
nsPEFs and everolimus induced melanoma cell apoptosis, cellular mitochondrial membrane potential (ΔΨm) loss and Ca^2+^ increase. (**a**) A375 cells were treated with everolimus for 48 h, followed by nsPEF treatment. Cell apoptosis was evaluated by FITC-Annexin V and PI staining using flow cytometry. The experiments repeated 3 times yielded similar results. Cells after nsPEF treatment were incubated with JC-1 or Fluo-3/AM at 37 °C for 30 min. (**b**) Loss of ΔΨm was detected using the fluorescent probe JC-1. Cells treated with 100 nM valinomycin were used as the positive control for decreased ΔΨm. (**c**) Intracellular Ca^2+^ level was quantified with the fluorescent dye Fluo-3/AM. Cells treated with 20 μM ionomycin were used as the positive control for intracellular Ca^2+^ level detection. The experiments repeated thrice yielded similar results. Values are means ± SD (n = 6). *P < 0.05 compared to control, ^#^P < 0.05 compared to everolimus single agent treatment.

Cell apoptosis was detected 24 h after nsPEF treatment. nsPEF energy-dependent apoptosis was observed in both cell lines, while everolimus alone could only slightly induce cell apoptosis compared to the control even when the drug concentration achieved 1 μM. We therefore examined whether the combination of everolimus and nsPEFs could further induce cell apoptosis in A375 cells. The results showed that although everolimus did not induce strong apoptosis, the combination of everolimus and nsPEFs exhibited a synergistic effect in inducing cell apoptosis (Fig. 3a).

### nsPEFs induced cellular ΔΨm loss and Ca^2+^ increase

Cellular ΔΨm loss and Ca^2+^ increase were detected after nsPEF treatment with JC-1 and Fluo-3 staining. For everolimus single agent, cellular ΔΨm loss and Ca^2+^ increase were detected after 24 h incubation. When ΔΨm decreases, JC-1 changes from red aggregates to green monomers. As shown in Fig. 3b, everolimus treatment could induce only a minor ΔΨm decrease, whereas nsPEFs led to a greater ΔΨm decrease compared to everolimus. Everolimus combined with nsPEFs exhibited significant synergism with increased green fluorescence intensity and decreased red fluorescence intensity, which indicated severe deaggregation of JC-1 aggregates and strong ΔΨm loss. Increased intracellular Ca^2+^ levels were detected immediately after nsPEF treatment and exhibited a pattern of dependence on nsPEF intensity (Fig. 3c).

### nsPEF plus everolimus treatment suppressed tumour growth *in vivo*

**Figure 4:**
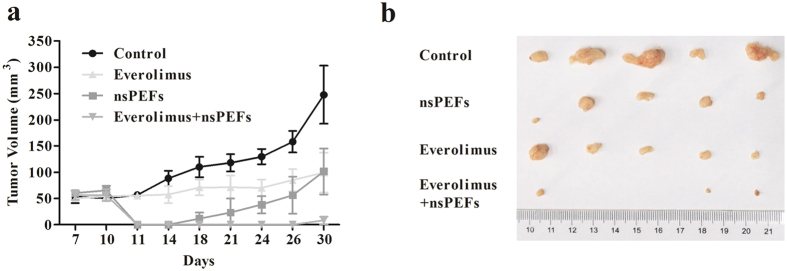
nsPEFs and everolimus synergistically suppressed tumour growth *in vivo*. (**a**) Tumour volume change in the 4 groups after treatment. Tumour size was measured every 3–4 days after treatment. Values are means ± SEM (n = 5 mice per group). (**b**) Tumours derived from 4 groups 30 days after subcutaneous injection.

We further investigated the effectiveness of everolimus and nsPEFs *in vivo*, A375 cells were used for establishment of orthotopic model. As shown in Fig. 4a and Supplementary Fig. S3, tumours of the control group showed steady growth after inoculation and started dramatic growth 24 days after inoculation. Single-agent everolimus induced controlled tumour growth; tumours remained almost the same in the first 2 weeks after treatment, and then started to grow rapidly after drug withdrawal. With nsPEF treatment alone, tumour size decreased significantly right after the pulse. However, the growth inhibition effect of nsPEFs was sustained only for 1 week; the tumours started to grow back on day 18. The tumour growth of the combination treatment group steadily decreased throughout the experimental period, and the tumours could not even be measured at 3 weeks after treatment, the tumours of 2 of 5 (40%) mice achieved complete regression. At the endpoint of the control group, all mice were sacrificed, and tumours were dissected and weighed. Both rapamycin and nsPEFs significantly decreased tumour growth, with an average tumour volume of 99.24 ± 37.65 mm^3^ (p = 0.019) and 101.57 ± 43.90 mm^3^ (p = 0.020), respectively, in comparison with 247.57 ± 55.21 mm^3^ of the control mice. The combination of rapamycin and nsPEFs improved the inhibitory effect, with an average tumour volume of 8.30 ± 7.43 mm^3^ (p = 0.001).

### nsPEFs and everolimus induced cell apoptosis and suppressed angiogenesis *in vivo*

**Figure 5:**
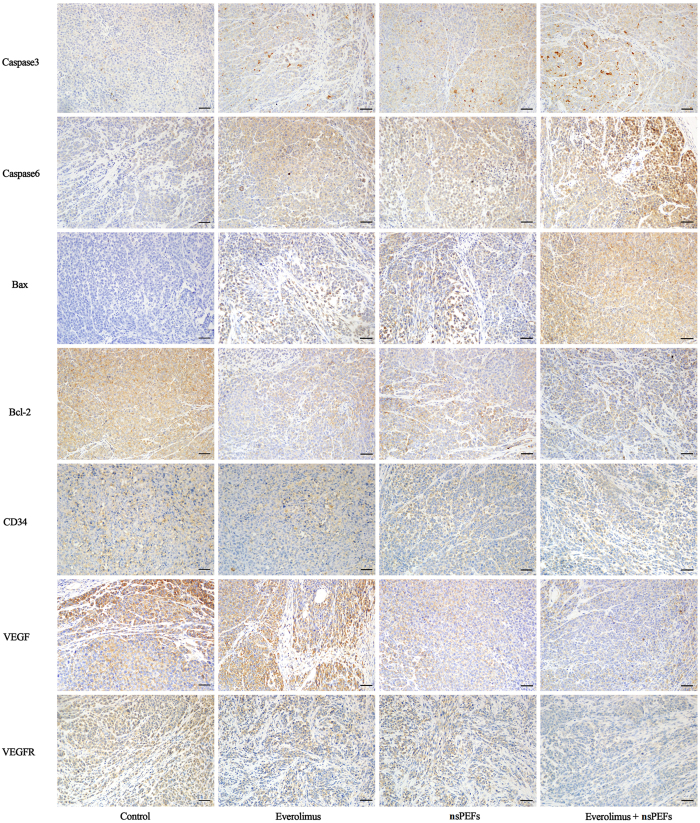
nsPEFs and everolimus induced cell apoptosis and decreased angiogenesis *in vivo*. Immunohistochemical staining of the apoptosis markers Bax, Bcl-2, cleaved caspase-3, and cleaved caspase-6, and angiogenesis-associated markers VEGF, VEGFR, and CD34 (original magnification, ×200).

To investigate whether nsPEFs alone or in combination with everolimus decrease melanoma tumour growth by promoting tumour cell apoptosis *in vivo*, we examined the markers related to cell apoptosis by immunohistochemistry. As shown in Fig. 5, compared to the control group, both nsPEF- and everolimus-treated tumours showed increased expression of caspase 3, caspase 6, and Bax, and decreased expression of Bcl-2. Combined treatment showed a stronger increase than that by everolimus or nsPEF treatment alone. VEGF and VEGFR are important mediators during angiogenesis in cancer^35^. The endothelial cell marker CD34 is generally used in the evaluation of angiogenesis. Tumours treated with either nsPEFs alone or everolimus alone exhibited decreased VEGF, VEGFR, and CD34 expression, while the combined treatment showed an increased inhibition of VEGF, VEGFR, and CD34 expression, indicating that nsPEFs and everolimus synergistically suppressed neovascular growth.

## Discussion

Metastatic melanoma is highly resistant to traditional chemotherapy and radiation therapy. The objective response rate of single-agent dacarbazine and temozolomide is less than 20%^36^. Over the past few years, significant progress in treating metastatic melanoma has been achieved with the development of individual targeted therapies and immunotherapies, but these therapeutic agents do not serve all patients because they are expensive and not available in some countries and regions. Exploring a safe, efficient, and affordable therapeutic strategy to improve the clinical condition of metastasis or local recurrence of malignant melanoma has important implications.

The conserved serine/threonine kinase mTOR forms 2 complexes, mTORC1 and mTORC2. The main accepted tumour ablation mechanism of everolimus is the dephosphorylation of S6K1 and 4EBP1 by inhibiting mTORC1, which results in cell apoptosis, G1/S cell cycle arrest, and angiogenesis suppression^37^,^38^,^39^,^40^. However, everolimus does not inhibit mTORC2, and the inhibition of mTORC1 leads to feedback activation of Akt and protects cancer cells from apoptosis^41^. In this study, we proposed that nsPEFs might enhance the anti-tumour efficacy of everolimus and might be used as a novel strategy for melanoma treatment. First, we evaluated the growth inhibitory effect of nsPEFs alone *in vivo* and *in vitro*. Melanoma cells were pulsed with high-energy electric fields. The results showed that nsPEFs could inhibit melanoma cell growth, and the effect was energy dependent. However, the inhibitory effect would reach a plateau when 30–40% surviving cells remained. This result was confirmed by an orthotopic model, where the tumour continued to shrink for 3 days after nsPEF treatment, and then robust proliferation appeared. This might be because nsPEFs stimulated the release of internally stored calcium. The calcium signal would induce resting G0 cells to re-enter the cell cycle and promote DNA synthesis at the G1/S transition^42^,^43^.

Our results demonstrated that the combination of everolimus and nsPEFs synergistically inhibited melanoma growth. Given that the effects of nsPEFs in combination with drugs have not been investigated in melanoma thus far, how to combine the 2 different tools to maximize the inhibition effect needs to be defined. The proliferation rates of 2 cell lines after different sequential orders of treatment were assessed, and both cell lines were more sensitive to nsPEFs following everolimus treatment. These results suggested that although everolimus could not kill the 2 melanoma cell lines, it could sensitize the cells to low-strength nsPEFs. This finding was similar with the results of our previous work in breast cancer, in which the chemotherapeutic drug gemcitabine was used in combination with nsPEFs, and treatment with gemcitabine before nsPEFs was the preferred sequential order to inhibit breast cancer cell viability and clonogenic survival^32^. In our *in vivo* study, nsPEFs showed a stronger retardation of tumour growth than everolimus since treatment initiation. However, after the nsPEF-induced scar healed, tumour growth in the nsPEF group accelerated, and there were no significant differences in tumour size and weight between the nsPEF and everolimus groups. The enhancement of efficacy *in vivo* was consistent with that *in vitro*, as the combination treatment displayed a better curative effect than that of all the other groups. Furthermore, the tumours of the 40% combination group achieved complete remission. It is possible that the synergistic effects of everolimus and nsPEFs could last for around a month, after which tumours would grow again. Tumours have been reported to grow again after 2 weeks’ regression with nsPEF treatment^28^. Although tumours start to grow again, they are still much smaller in size, which would make enable other approaches to be applied, thereby increasing the chances of achieving complete remission.

In the combination treatment group, cellular apoptosis was observed, with elevated expression of Bax, caspase 3, and caspase 6 and decreased expression of Bcl-2, indicating that nsPEFs and everolimus disturbed the balance between pro-apoptosis and anti-apoptosis signals and triggered apoptosis *in vivo*. The consequent DNA damage may also contribute to tumour growth inhibition. DNA damage has been observed in nsPEF-treated murine fibrosarcoma and melanoma. DNA double-stranded breaks and chromosome condensation appear shortly after nsPEF treatment^29^,^44^. Another widely accepted mechanism of tumour growth inhibition by nsPEF is by the suppression of angiogenesis. nsPEFs have been demonstrated to be able to inhibit the growth of tumour blood vessels and blood supply around tumours^28^,^45^. In our study, VEGF, VEGFR, and CD34 expression was strongly suppressed after treatment, indicating that the blood supply of tumours was inhibited.

It has been recognized that multiple cellular organs respond to nsPEF treatment. Our study showed that nsPEFs and everolimus induced cell apoptosis, accompanied with ΔΨm loss and increased Ca^2+^ levels. nsPEFs can trigger a rapid decrease in ΔΨm in multiple cell lines, including B16F10 murine melanoma and E4 squamous cells^46^,^47^. The rapid change in ΔΨm leads to the release of pro-apoptotic factors like cytochrome c and then contributes to caspase-dependent apoptosis^48^,^49^. nsPEFs are reported to trigger cytoplasmic Ca^2+^ increase almost immediately after treatment, causing both endoplasmic reticulum Ca^2+^ release and extracellular Ca^2+^ influx^50^,^51^,^52^. Increased Ca^2+^ levels can also induce cell death. Morotomi-Yano *et al*. found that nsPEFs could induce the necrosis of HeLa S3 cells in a Ca^2+^ -dependent manner in the presence of extracellular calcium, whereas apoptosis was induced in the absence of extracellular calcium^53^.

In summary, our study demonstrated that the combined treatment of nsPEFs with everolimus could synergistically decrease melanoma cell growth, promote cell apoptosis, and inhibit angiogenesis. This finding suggested that as a promising physical sensitizer, nsPEFs provide valuable impetus to molecular targeting drugs, and that combination therapy using nsPEFs and everolimus can be optimized for further clinical treatment of advanced melanoma.

## Materials and Methods

### Cell lines and cell culture

Human melanoma cell lines A375 and A875 were obtained from the Cell Resource Center, IBMS, CAMS/PUMC (Beijing, China). M21 was purchased from National Institutes for Food and Drug Control (Beijing, China). The 293T cell line was purchased from Leibniz Institute (DSMZ, Braunschweig, Germany). All 4 cell lines were cultured in DMEM (Gibco, Invitrogen, Grand Island, NY, USA) supplemented with 10% FBS (Gibco), 2 mM l-glutamine, and 1% penicillin-streptomycin (Gibco). WM-115 was purchased from ATCC (Manassas, VA, USA) and cultured in EMEM (Gibco, Invitrogen, Grand Island, NY, USA) supplemented with 10% FBS (Gibco), 2 mM l-glutamine, and 1% penicillin-streptomycin (Gibco).

### Animals

Four- to five-week-old BALB/c (nu/nu) female mice were purchased and maintained under pathogen-free conditions in the Peking University Laboratory Animal Center. All animal experiments were carried out in accordance with the National Institutes of Health guide for the care and use of laboratory animals (NIH Publications No. 8023, revised 1978), and were approved by the medical ethics committee of the Peking University Cancer Hospital & Institute. A375 cells (5 × 10^6^) in 100 μL PBS were inoculated subcutaneously into flanks. All mice were classified into 4 groups when tumours reached 5–7 mm in diameter: group I (control group), treated with sham pulse and 0.9% saline; group II, treated with 100 pulses of nsPEFs at 30 kV/cm for 100 ns; group III, treated with everolimus (4 mg/kg of body weight) by oral gavage daily for 2 weeks; and group IV, treated with everolimus (4 mg/kg of body weight for 2 weeks) in combination with nsPEFs (100 pulses of 30 kV/cm, 100 ns). nsPEFs were delivered on the first day of everolimus administration. Tumour sizes and mouse weights were measured twice a week. Each tumour was measured with a calliper, and tumour volume was calculated by the following formula: volume = 0.52 × *a* × *b*^2^, where *a* = longest diameter and *b* = shortest diameter. When the tumours of the control group were >1.5 cm in diameter, all mice were sacrificed by exposure to an atmosphere highly enriched with CO_2_, and the xenografts were excised and weighed.

### Chemicals and antibodies

Everolimus was purchased from Selleckchem Co. (Shanghai, China). For the *in vivo* experiments, a microemulsion was freshly diluted in a vehicle of 5% glucose at an administration volume of 4 mg/kg. The cleaved caspase antibody sampler kit, and antibodies against phospho-mTOR, phospho-S6RP, phospho-4EBP1, mTOR, S6RP, and 4EBP1 were purchased from Cell Signaling Technology (Beverly, MA, USA). Bcl-2, Bax, VEGF, VEGF receptor (VEGFR), and CD34 antibodies were purchased from Bioworld Technology (Minneapolis, MN, USA).

### nsPEF application

**Figure 6:**
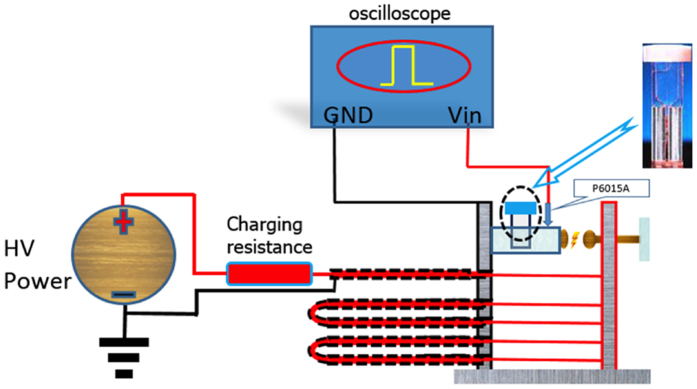
Schematic diagram of the nsPEF generator.

We established a transmission line–based nsPEF generator, as shown in the schematic diagram in Fig. 6. The pulse duration was fixed at 100 ns, and the intensity of electric fields varied from 5 to 40 kV/cm. For the *in vitro* study, cells were harvested and suspended in a cuvette (BTX Co., San Diego, CA, USA) with a 2-mm gap in complete culture medium. All cells were exposed to 25 pulses at electric fields between 10 kV/cm to 30 kV/cm. For the *in vivo* study, tumours were fixed between the 2 clamps of the applicator to accept nsPEF treatment. The applicator was made of insulating materials with 2 copper strips in opposite positions inside the clamp, and an electric field was generated between the 2 copper strips. Throughout the treatment process, all mice were maintained under sodium pentobarbital anaesthesia.

### Cell proliferation and apoptosis assays

Cell viability was measured by the CellTiter-Glo luminescent cell viability assay kit (Promega, Madison, WI, USA) according to the manufacturer’s instructions, and luminescence was measured using a BioTek Synergy 2 luminometer (BioTek, Winooski, VT, USA). Cell apoptosis was evaluated by FITC-Annexin V and PI staining (Merck, Darmstadt, Germany), and stained samples were analysed by a FACS flow cytometer (Becton Dickinson, USA).

### Western blotting

When cells reached 70–80% confluency, they were starved overnight and treated with control vehicle (DMSO) or 1 μM everolimus for 1 h. Cells were lysed using PhosphoSafe extraction reagent (EMD Millipore, Billerica, MA, USA) and subjected to NuPAGE (Invitrogen, Carlsbad, CA, USA) according to standard procedures.

### Mitochondrial membrane potential and intracellular Ca^2+^ detection

Loss of mitochondrial membrane potential (ΔΨm) was detected using the fluorescent probe JC-1 (Beyotime, Shanghai, China), and intracellular Ca^2+^ level was quantified with the fluorescent dye Fluo-3/AM (Beyotime). For nsPEF signle agent and combination treatment, cellular ΔΨm loss and Ca^2+^ increase were detected after nsPEF treatment and incubated with JC-1 or Fluo-3/AM at 37 °C for 30 min. For everolimus single agent, cellular ΔΨm loss and Ca^2+^ increase were detected after 24 h incubation. Next, cells were washed with PBS and analysed with a fluorescence plate reader.

### Lenti-GFP infection and *in vivo* fluorescence imaging

The day before transfection, 293 T cells were seeded at 5 × 10^6^ cells per 100-mm dish. The next day, GFP lentiviral vector, psPAX2 packaging plasmid, and pMD2.G envelope vector were added to 293T cells using the FuGENE 6 transfection reagent (Promega) according to the manufacturer’s instructions. Virus-containing supernatants were collected 48 h post transfection. A375 cells were incubated in the virus-containing supernatants overnight and replaced in fresh culture medium. A375-GFP cells were inoculated subcutaneously into the flanks of mice, and tumour images before and after nsPEF treatment were monitored. Mice were anesthetized with 2.5% isoflurane, and the GFP activity was localized and quantified using an IVIS 200 *in vivo* optical imaging system (Xenogen Corp., Alameda, CA, USA). Images were obtained with an excitation wavelength of 465 nm and emission wavelength ranging from 500 to 540 nm. Imaging processing and analysis were performed with Living Image 3.0 software (Caliper Life Sciences, Hopkinton, MA, USA).

### Immunohistochemistry assay

Tumours were fixed in 4% paraformaldehyde, embedded with paraffin, and cut into 4–5-μm sections. The slides were deparaffinised and rehydrated in PBS. Antigen retrieval was performed by microwaving in citrate buffer (pH 6) for 10 min. Endogenous peroxidase was blocked by 3% hydrogen peroxide for 10 min. Slides were blocked with 10% goat serum for 40 min. Primary antibodies were diluted to 1:500 to 1:1000 and incubated at 4 °C overnight. Secondary antibodies were added and incubated at room temperature for 1 h. DAB was used as a chromogen, and slides were sealed with balsam.

### Statistical analysis

Statistical analyses were performed using SPSS 16.0 software. Values of mean ± SD were determined, and differences among groups were determined by Student’s *t* test or ANOVA. Statistical significance was considered at p < 0.05.

## Additional Information

**How to cite this article:** Dai, J. *et al*. Nanosecond Pulsed Electric Fields Enhance the Anti-tumour Effects of the mTOR Inhibitor Everolimus against Melanoma. *Sci. Rep.* **7**, 39597; doi: 10.1038/srep39597 (2017).

**Publisher's note:** Springer Nature remains neutral with regard to jurisdictional claims in published maps and institutional affiliations.

## Supplementary Material

Supplementary Information
