# Supplementary material for: Nanosecond Pulsed Electric Fields Enhance the Anti-tumour Effects of the mTOR Inhibitor Everolimus against Melanoma
Source: Sci Rep. 2017 Jan 5;7:39597. doi: 10.1038/srep39597 (PMC5215571; doi:10.1038/srep39597)

Nanosecond Pulsed Electric Fields Enhance the Anti-tumour Effects of the mTOR Inhibitor Everolimus against Melanoma

Jie Dai[1,#], Shan Wu[2,#], Yan Kong[1,#], Zhihong Chi[1], Lu Si[1], Xinan Sheng[1], Chuanliang Cui[1], Jing Fang[2], Jue Zhang[2,*], Jun Guo[1,*]

[1] Key Laboratory of Carcinogenesis and Translational Research (Ministry of Education), Department of Renal Cancer and Melanoma, Peking University Cancer Hospital & Institute, Beijing, China

[2] College of Engineering, Academy for Advanced Interdisciplinary Studies, Peking University, Beijing, China

Footnotes:

[#] J. Dai, S. Wu and Y. Kong contributed equally to this work.

[*] **Corresponding authors**: Jue Zhang, Ph.D., College of Engineering, Peking University, 5 Yiheyuan Rd, Beijing, 100871.

Telephone: +861062755036. Email: zhangjue@pku.edu.cn

Jun Guo, M.D., Ph.D., Department of Renal Cancer and Melanoma, Peking University Cancer Hospital & Institute, 52 Fucheng Road, Haidian District, Beijing 100142, China.

Telephone: +861088196317. Email: guoj307@126.com.

Supplementary Table S1. Parameters of nsPEF treatment showed in figure 1

Parameters	0E	1E	1.5E	1.6E	2.2E	2.4E	3.2E	3.6E
Electric field strength (kV/cm)	0	20	30	20	30	20	20	30
Pulse duration (ns)	0	100	100	100	100	100	100	100
Number of pulses	0	10	10	25	25	50	100	50

Figure legends

Supplementary Figure S1. nsPEF treatment inhibited melanoma cell growth *in vivo* and *in vitro*. Effects of nsPEFs with high energy input in (a) M21 and (b) WM-115 cells. Pulse duration, 100 ns; electric field strength, 20–30 kV/cm; number of pulses delivered according to different energy levels, 10–100. The energy input of nsPEFs was calculated as energy input = $(E^2 \times D^2 \times W \times N)/(R \times M)$, where E is the electric field strength (20–30 kV/cm); D, gap between electrodes (here, 2 mm); W, pulse duration (here, 100 ns); N, number of pulses (10–100 pulses); R, resistance in the cuvette with cells and suspending medium; and M, mass of the suspension in the cuvette. The specific parameters are listed in Supplementary Table S1.

Supplementary Figure S2. Effect of everolimus on activation of mTOR signalling in melanoma cells. Cells were starved overnight and treated with control vehicle (DMSO) or 1 μM everolimus for 1 h, then cells were lysed, and the activation of indicated molecules were examined by western blotting. Lane 1-2 were control of A375 and A875 cells, lane 3 was A375 cells treated with everolimus, and lane 4 was A875 cells treated with everolimus.

Supplementary Figure S3. Individual tumour plots of tumour volume change after treatments.

Dai J. et al., Supplementary Figure S1

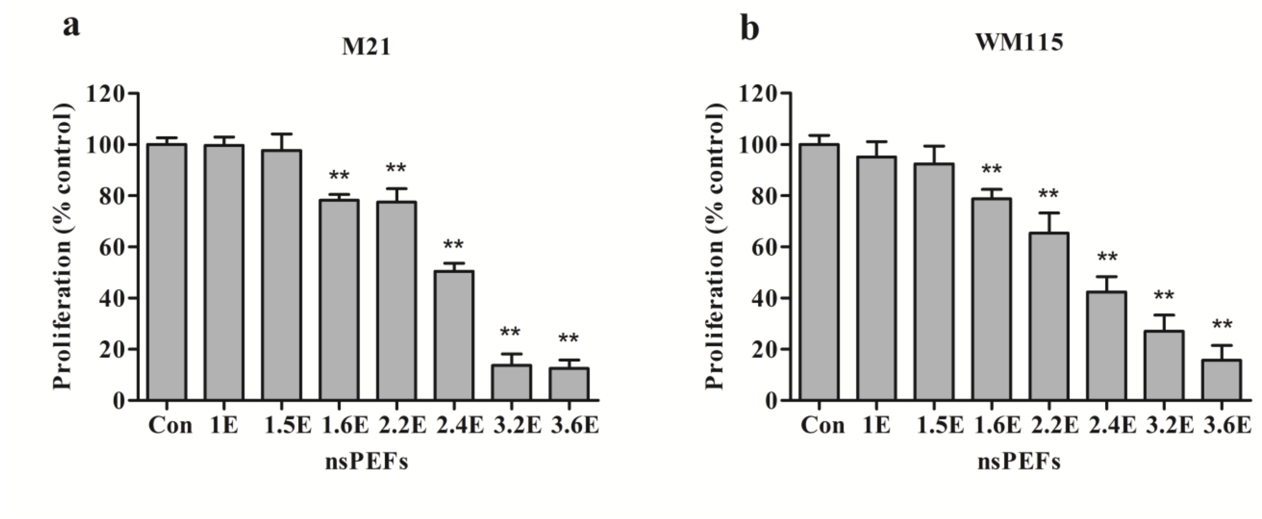

Dai J. et al., Supplementary Figure S2

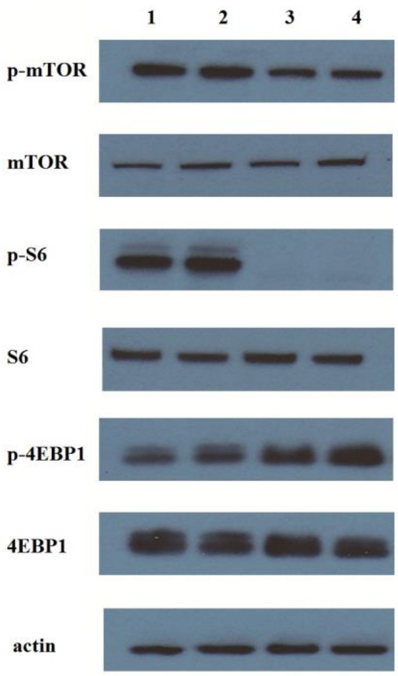

Dai J. et al., Supplementary Figure S3

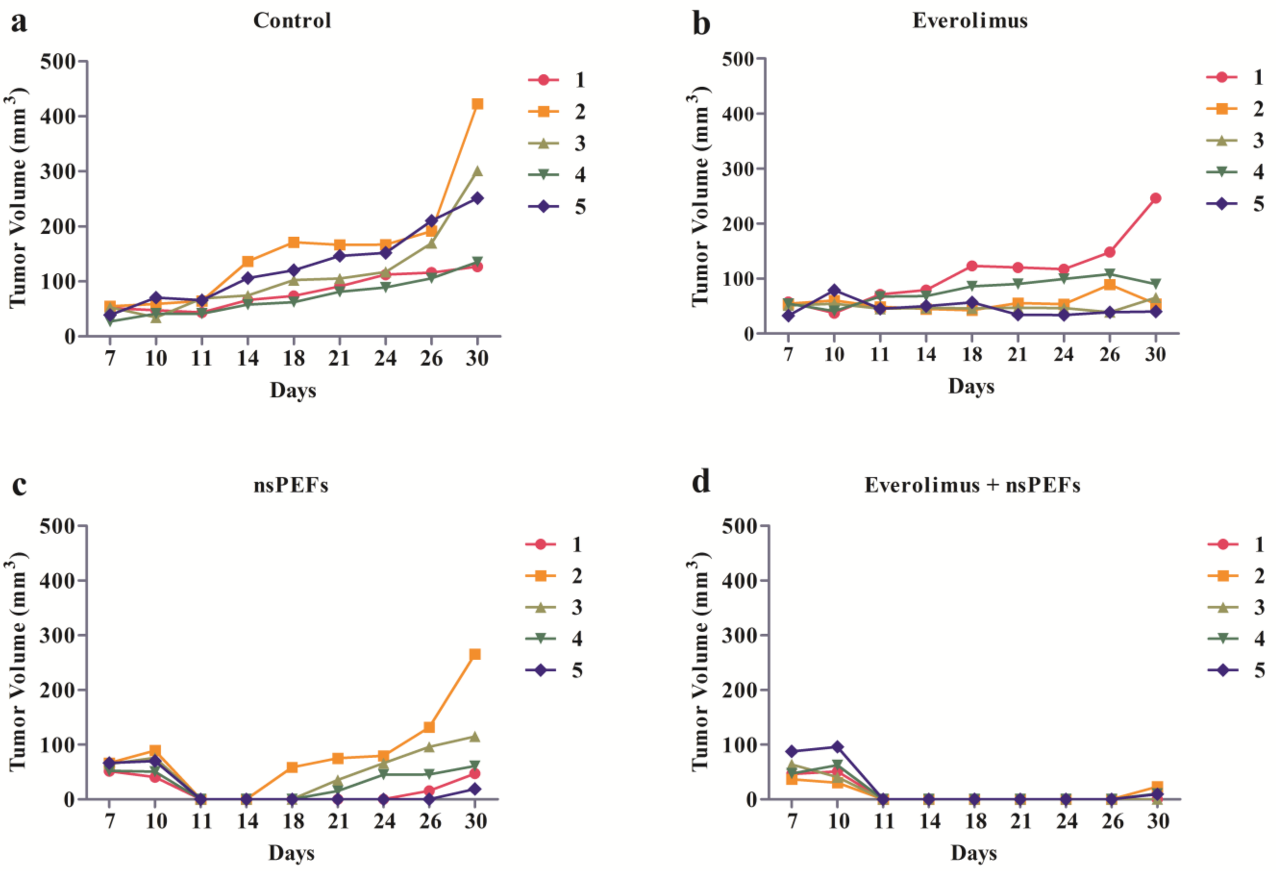

Supplement: Supplementary Information [file srep39597-s1.pdf]
